# Diurnal Variations in Serum Uric Acid, Xanthine, and Xanthine Oxidoreductase Activity in Male Patients with Coronary Artery Disease

**DOI:** 10.3390/nu15204480

**Published:** 2023-10-23

**Authors:** Megumi Shimizu, Ryo Naito, Akihiro Sato, Sayaki Ishiwata, Shoichiro Yatsu, Jun Shitara, Hiroki Matsumoto, Azusa Murata, Takao Kato, Shoko Suda, Masaru Hiki, Masanari Kuwabara, Takayo Murase, Takashi Nakamura, Takatoshi Kasai

**Affiliations:** 1 Department of Cardiovascular Biology and Medicine, Juntendo University Graduate School of Medicine, Tokyo 113-8421, Japan; megumi-s@juntendo.ac.jp (M.S.); ak-sato@juntendo.ac.jp (A.S.); s-ishiwata@juntendo.ac.jp (S.I.); syatsu@juntendo.ac.jp (S.Y.); jshitara@juntendo.ac.jp (J.S.); hmatsumo@juntendo.ac.jp (H.M.); azmurata@juntendo.ac.jp (A.M.); tkatou@juntendo.ac.jp (T.K.); ssuda@juntendo.ac.jp (S.S.); ma-hiki@juntendo.ac.jp (M.H.); tkasai@juntendo.ac.jp (T.K.); 2 Keiyu Orthopedic Spine and Joint Hospital, Tokyo 120-0015, Japan; 3 Cardiovascular Respiratory Sleep Medicine, Juntendo University Graduate School of Medicine, Tokyo 113-8421, Japan; 4 Intensive Care Unit and Department of Cardiology, Toranomon Hospital, Tokyo 105-8470, Japan; kuwamasa728@gmail.com; 5 Sanwa Kagaku Kenkyusho Co., Ltd., Inabe 511-0406, Japan; ta_murase@skk-net.com (T.M.); ta_nakamura@mb4.skk-net.com (T.N.); 6 Sleep and Sleep Disordered Breathing Center, Juntendo University Hospital, Tokyo 113-8431, Japan

**Keywords:** uric acid, xanthine, xanthine oxidoreductase, purine metabolism, cardiovascular disease, sleep-disordered breathing

## Abstract

Hyperuricemia is influenced by diet and can cause gout. Whether it is a potential risk factor for cardiovascular disease (CVD) remains controversial, and the mechanism is unclear. Similar to CVDs, gout attacks occur more frequently in the morning and at night. A possible reason for this is the diurnal variation in uric acid (UA), However, scientific data regarding this variation in patients with CVD are not available. Thus, we aimed to investigate diurnal variations in serum levels of UA and plasma levels of xanthine, hypoxanthine, and xanthine oxidoreductase (XOR) activity, which were measured at 18:00, 6:00, and 12:00 in male patients with coronary artery disease. Thirty eligible patients participated in the study. UA and xanthine levels significantly increased from 18:00 to 6:00 but significantly decreased from 6:00 to 12:00. By contrast, XOR activity significantly increased both from 18:00 to 6:00 and 6:00 to 12:00. Furthermore, the rates of increase in UA and xanthine levels from night to morning were significantly and positively correlated. In conclusion, UA and xanthine showed similar diurnal variations, whereas XOR activity showed different diurnal variations. The morning UA surge could be due to UA production. The mechanism involved XOR activity, but other factors were also considered.

## 1. Introduction

**Figure 1:**
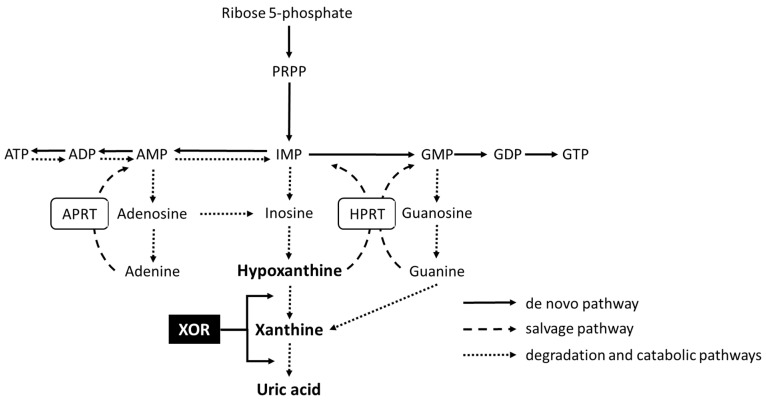
Purine metabolism. ADP—adenosine diphosphate; AMP—adenylic acid; APRT—adenine phosphoribosyltransferase; ATP—adenosine triphosphate; GDP—guanosine diphosphate; GMP—guanosine monophosphate; GTP—guanosine triphosphate; HPRT—hypoxanthine phosphoribosyltransferase; IMP—inosinic acid; PRPP—phosphoribosyl diphosphate; XOR—xanthine oxidoreductase.

Diet and sleep play important roles in the development of cardiovascular disease (CVD) [1,2]. Disordered dietary habits lead to hypertension, dyslipidemia, and diabetes, which are obvious risk factors for CVDs [3,4,5,6,7,8,9,10]. Excessive intake of a purine-rich diet causes hyperuricemia [11,12]. Other factors, such as obesity due to poor diet and increased alcohol consumption, also increase the incidence of hyperuricemia [13,14]. Some large epidemiological investigations have established a correlation between hyperuricemia and cardiovascular risk in the general populace [15,16]. This correlation has also been observed among individuals diagnosed with hypertension [17]. Some investigations have asserted that serum uric acid (UA) levels is an independent risk factor for CVD [18]. In contrast, other studies have deduced that there is merely an association between serum UA levels and additional risk factors, such as hypertension, renal issues, elevated lipoprotein levels, and the utilization of diuretic agents [19]. Thus, Hyperuricemia has been reported to be a predictor of CVD; however, whether it is an independent risk factor remains controversial. Previous studies have suggested an association between sleep and UA. For instance, patients with obstructive sleep apnea, which is associated with an increased risk of CVD and overall cardiovascular mortality [20,21], tend to have higher serum UA levels [22]. Our previous study showed that the severity of nocturnal hypoxia is positively correlated with xanthine oxidoreductase (XOR) activity, which is involved in purine metabolism (Figure 1). Hypoxia enhances oxidative stress through XOR activity and increases UA production, which suggests a relationship between sleep and UA [23]. Hyperuricemia is the most important risk factor for the development of gout. The development of gout is influenced by several risk factors, including increased age, genetic predisposition, and diet [24]. In addition, elevated UA levels can arise from both increased production and reduced excretion of UA [25]. Furthermore, gout attacks are more common at night and at dawn [26], when the incidence of CVD is also common [27,28]. Diurnal variation in UA is one of the mechanisms involved. The diurnal variation in serum UA levels in normal subjects, patients with diabetes, and patients with multiple sclerosis not only increases from night to morning but also decreases from morning to noon [29,30]. Elucidation of the mechanisms underlying such diurnal variations may help prevent the onset of hyperuricemia, gout, and possibly CVD. However, no scientific data regarding diurnal variations in UA levels in patients with CVD are available. In addition, to understand the detailed mechanisms of such diurnal variations in serum UA levels, investigations of other metabolites of the XOR pathway are important. Therefore, we aimed to investigate diurnal variations in serum levels of UA and plasma levels of xanthine, hypoxanthine, and XOR activity in purine metabolism in male patients with coronary artery disease (CAD).

## 2. Materials and Methods

### 2.1. Patients

This is a prospective observational study enrolling male patients with CAD. They were admitted for elective percutaneous coronary intervention at Juntendo University Hospital between June 2016 and November 2017. In this study, we established inclusion criteria that restricted participation to male patients, as UA levels vary significantly between males and females and UA levels also exhibit sex-specific differences as a CAD risk marker [31]. We excluded patients who were currently receiving treatment for hyperuricemia, taking medications that affect serum UA levels (such as diuretics), and patients with a history of treated sleep apnea, heart failure, chronic pulmonary disease, cancer, or chronic kidney disease (CKD). The Ethics Committee of Juntendo University Hospital approved the study protocol (Approval no.15-179), which abides by the Declaration of Helsinki. Informed consent was obtained from all individuals.

### 2.2. Height, Weight, and Blood Pressure

The calculation of body mass index involved dividing the square of height in meters by weight in kilograms. Blood pressure was measured using a fully automated oscillometric sphygmomanometer when the patients woke up. Hypertension was defined as being under treatment with antihypertensive agents or having systolic blood pressure ≥ 140 mmHg and/or diastolic blood pressure ≥ 90 mmHg. CKD was defined as an estimated glomerular filtration rate < 60 mL/min/1.73 m^2^ [32]. Dyslipidemia was defined as triglyceride levels ≥ 150 mg/dL, low-density lipoprotein cholesterol levels ≥ 140 mg/dL, high-density lipoprotein cholesterol levels ≤ 40 mg/dL, or under treatment with lipid-lowering agents. Diabetes mellitus was defined as hemoglobin A1c (National Glycohemoglobin Standardization Program) levels ≥ 6.5% and fasting blood glucose levels ≥ 126 mg/dL or under treatment with oral antidiabetic agents or insulin.

### 2.3. Blood Samples

We measured the serum levels of UA and plasma levels of xanthine, hypoxanthine, and XOR activity at three time points: 18:00, 6:00, and 12:00. In detail, samples of all patients were taken on consecutive days at 18:00 on the third day and at 6:00 and 12:00 on the fourth day of admission. Additionally, samples were taken before each meal. Serum levels of total cholesterol, triglycerides, and high-density lipoprotein cholesterol were measured using standard enzymatic methods, and low-density lipoprotein cholesterol levels were calculated using the Friedewald formula (non-high-density lipoprotein cholesterol level−triglycerides/5). Plasma glucose, hemoglobin A1c, and creatinine levels were measured using standard methods.

### 2.4. Hypoxanthine and Xanthine

The plasma levels of purine degradation products, hypoxanthine, and xanthine were measured according to the following method: one hundred microliters of plasma samples was mixed with 300 μL of methanol containing [^13^C_3_, ^15^N] hypoxanthine and [^13^C_2_, ^15^N_2_] xanthine as internal standards. The mixtures were centrifuged at 3000× *g* for 15 min at 4 °C. Subsequently, 30 μL of the supernatant was transferred to new tubes, diluted with 120 μL of distilled water, and analyzed using liquid chromatography–triple quadrupole mass spectrometry (LC/TQMS). The concentrations of hypoxanthine and xanthine in the plasma were quantified from calibration curves obtained using calibration standards [33].

### 2.5. XOR Activity

The XOR activity assay was conducted using a stable isotope-labeled substrate and LC/TQMS (Sanwa Kagaku Kenkyusho Co., Ltd., Inabe, Japan) [34]. In detail, plasma aliquots were collected and promptly stored at −80 degrees Celsius until analysis. Plasma XOR activity was determined using a combination of [^13^C_2_,^15^N_2_]xanthine and LC/TQMS as previously described [34]. This assay demonstrated linearity in the calibration curve within the range of 4 and 4000 nmol/L (R^2^ > 0.995), with a lower limit of quantitation of 4 nmol/L. This lower limit corresponds to an XOR activity of 6.67 pmol/h/mL of plasma.

### 2.6. Statistical Analysis

In this study, for continuous variables, values are presented as mean ± standard deviation or median (interquartile range), and for nominal variables, they are expressed as numbers (%). Due to the skewed nature of XOR activity, all statistical analyses employed the natural log-transformed XOR activity (log XOR). The sample size calculation relied on data from the initial five subjects, as no prior relevant studies were available. These data revealed mean values and standard deviations of changes in UA levels as 0.1 mg/dL and 0.19, respectively. With a significance level of 0.05 and 80% power, the calculated sample size was 30. A paired *t*-test was used to compare the UA, xanthine, hypoxanthine, and XOR activities at 18:00, 6:00, and 12:00. The Bonferroni correction was used after the paired *t*-test. Repeated-measures analysis of variance (ANOVA) was used to assess diurnal variation at three time points (18:00, 6:00, and 12:00). The percentage changes in UA, xanthine, and hypoxanthine from 18:00 to 6:00 and 6:00 to 12:00 are expressed as ΔUA (18:00–6:00), ΔUA (6:00–12:00), Δxanthine (18:00–6:00), Δxanthine (6:00–12:00), Δhypoxanthine (18:00–6:00), and Δhypoxanthine (6:00–12:00), respectively. Pearson correlation analysis was used to compare ΔUA, Δxanthine, and Δhypoxanthine values. Analyses were performed using JMP (version 5.0; SAS Institute, Cary, NC, USA).

## 3. Results

### 3.1. Baseline Patient Characteristics

Overall, 30 patients were enrolled in this study. Table 1 shows the baseline patient characteristics. The mean age was 67.6 years, and the mean body mass index was 24.5 kg/m^2^. The prevalence rates of hypertension, dyslipidemia, diabetes mellitus, and current smoking were 73%, 90%, 53%, and 27%, respectively. A total of 53% were taking angiotensin-converting enzyme inhibitor/angiotensin Ⅱ receptor blocker, 70% were taking β blockers, and 93% were taking statins.

### 3.2. Diurnal Variations in Serum UA and Plasma Xanthine, Hypoxanthine, and XOR Activity

**Figure 2:**
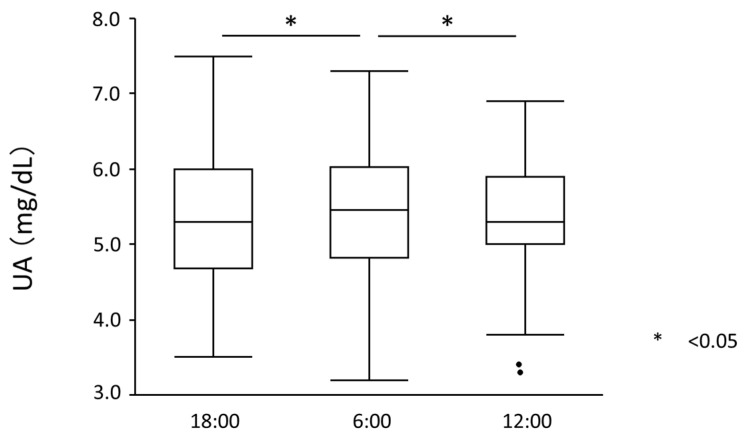
Serum UA levels. Serum UA levels significantly increased from 18:00 to 6:00 (5.27 ± 0.92 mg/dL to 5.41 ± 0.98 mg/dL, *p* = 0.012) and significantly decreased from 6:00 to 12:00 (5.41 ± 0.98 mg/dL to 5.31 ± 0.94 mg/dL, *p* = 0.018). UA—uric acid. Dots outside the boxplots are outliers which are 1.5 times the interquartile range less than the first quartile or 1.5 times the interquartile range greater than the third quartile.

**Figure 3:**
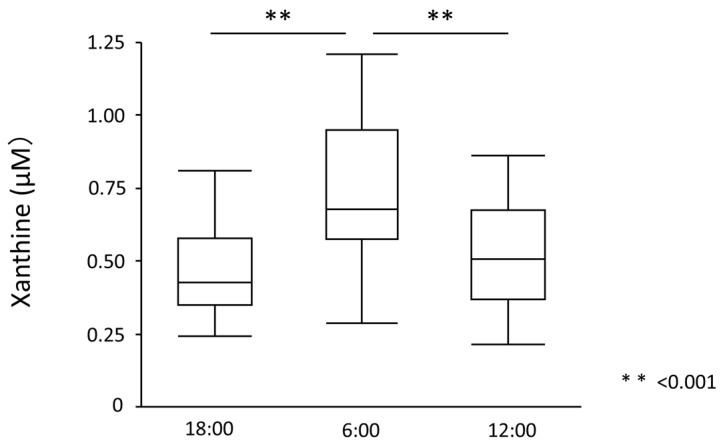
Plasma xanthine levels. Plasma xanthine levels significantly increased from 18:00 to 6:00 (0.46 ± 0.15 mg/dL to 0.74 ± 0.23 mg/dL, *p* < 0.001) and significantly decreased from 6:00 to 12:00 (0.74 ± 0.23 mg/dL to 0.52 ± 0.18 mg/dL, *p* < 0.001).

**Figure 4:**
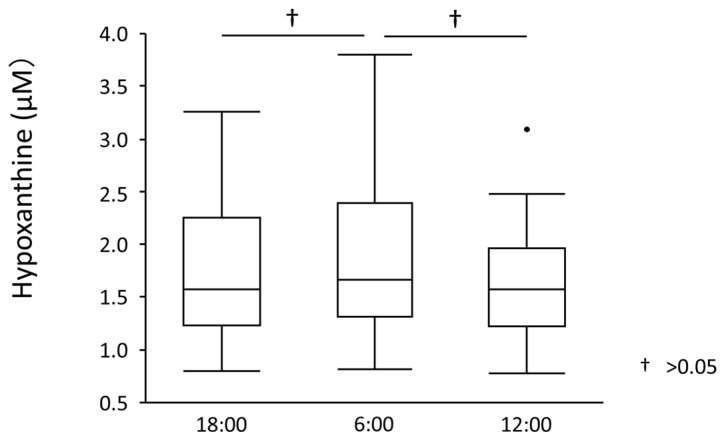
Plasma hypoxanthine levels. Plasma hypoxanthine levels did not change significantly between 18:00 and 12:00 or between 12:00 and 6:00 (1.72 ± 0.61 mg/dL to 1.86 ± 0.69 mg/dL, *p* = 0.923) (1.86 ± 0.69 to 1.61 ± 0.53 mg/dL, *p* = 0.113), and no diurnal variation was observed. Dots outside the boxplots are outliers which are 1.5 times the interquartile range less than the first quartile or 1.5 times the interquartile range greater than the third quartile.

**Figure 5:**
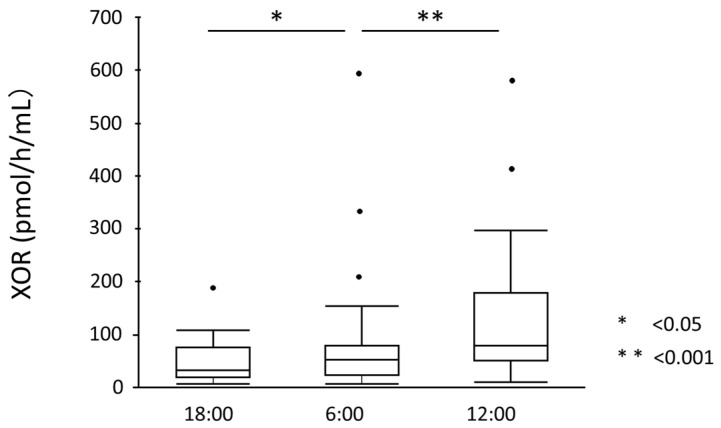
Plasma XOR activity. Plasma XOR activity significantly increased both from 18:00 to 6:00 (47.91 ± 39.57 mg/dL to 82.42 ± 116.0 mg/dL, *p* = 0.009) and from 6:00 to 12:00 (82.42 ± 116.0 mg/dL to 128.29 ± 123.57 mg/dL, *p* < 0.001). Dots outside the boxplots are outliers which are 1.5 times the interquartile range less than the first quartile or 1.5 times the interquartile range greater than the third quartile.

Serum UA levels significantly increased from 18:00 to 6:00 (5.27 ± 0.92 mg/dL to 5.41 ± 0.98 mg/dL, *p* = 0.012) but significantly decreased from 6:00 to 12:00 (5.41 ± 0.98 mg/dL to 5.31 ± 0.94 mg/dL, *p* = 0.018) (Figure 2). UA showed diurnal variation between 18:00 and 12:00 (*p* for ANOVA = 0.010). Similarly, plasma xanthine levels significantly increased from 18:00 to 6:00 (0.46 ± 0.15 mg/dL to 0.74 ± 0.23 mg/dL, *p* < 0.001) but significantly decreased from 6:00 to 12:00 (0.74 ± 0.23 mg/dL to 0.52 ± 0.18 mg/dL, *p* < 0.001) (Figure 3). Plasma xanthine levels showed diurnal variation between 18:00 and 12:00 (*p* for ANOVA < 0.001). Plasma hypoxanthine levels did not significantly change between 18:00 and 12:00 or between 12:00 and 6:00 (1.72 ± 0.61 mg/dL to 1.86 ± 0.69 mg/dL, *p* = 0.923) (1.86 ± 0.69 to 1.61 ± 0.53 mg/dL, *p* = 0.113), and no diurnal variation was observed (Figure 4). On the other hand, plasma XOR activity significantly increased both from 18:00 to 6:00 (47.91 ± 39.57 mg/dL to 82.42 ± 116.0 mg/dL, *p* = 0.009) and 6:00 to 12:00 (82.42 ± 116.0 mg/dL to 128.29 ± 123.57 mg/dL, *p* < 0.001) (Figure 5). XOR activity showed a diurnal variation between 18:00 and 12:00 (*p* for ANOVA < 0.001).

### 3.3. Formatting of Mathematical Components’ Correlation between ΔUA and Δxanthine and Δhypoxanthine

As the diurnal variations in UA and xanthine were similar, we evaluated the association between ΔUA and Δxanthine and Δhypoxanthine at the respective time points (18:00–6:00 and 6:00–12:00). From 18:00 to 6:00, a significant positive association was found between ΔUA and Δxanthine (r = 0.42, *p* = 0.020) and Δhypoxanthine (r = 0.37, *p* = 0.040) (Table 2). From 6:00 to 12:00, no association was found between ΔUA and Δxanthine or Δhypoxanthine (Table 3). The association between ΔXanthine and Δhypoxanthine was significantly positively correlated in both time periods from 18:00 to 6:00 (r = 0.73, *p* < 0.001) and 6:00 to 12:00 (r = 0.47, *p* < 0.01).

## 4. Discussion

The present study demonstrates that serum UA, plasma xanthine, and XOR activity showed diurnal variation in male patients with CAD. Our previous study showed that UA levels significantly increased from night to morning in male patients with CAD. In addition, we demonstrate that UA levels significantly decreased from 6:00 to 12:00 in this study. Previous studies have reported that UA levels show similar diurnal variations in healthy subjects, patients with diabetes, and patients with multiple sclerosis. In healthy individuals, UA oscillates with gradually increasing concentrations throughout the night, peaking in the morning [35]. In another study on healthy subjects and patients with diabetes, the mean serum urate level between 08:00 and 09:00 was higher than that observed between 17:00 and 18:00 [29]. In patients with multiple sclerosis, blood samples were taken at 3 h intervals over a 24 h period, and UA levels were measured, showing a peak from night to morning and a fall from morning to evening [30]. Our findings in male patients with CAD are also consistent with these results.

Furthermore, xanthine showed a diurnal variation similar to UA. By contrast, hypoxanthine showed no diurnal variation. To the best of our knowledge, this study is the first to demonstrate diurnal variations in xanthine. UA is the end product of purine metabolism in humans. Purine metabolism is a complicated mechanism consisting of the de novo pathway, salvage pathway, and degradation (Figure 1). In the de novo pathway, phosphoribosyl diphosphate is synthesized from ribose 5-phosphate and converted to inosinic acid (inosine monophosphate, IMP) through a 10-step reaction. IMP is converted to adenosine monophosphate (AMP) and guanylic acid (guanosine monophosphate, GMP). AMP forms adenosine, which is then deaminated to inosine. Inosine is then converted to hypoxanthine by purine nucleoside phosphorylase. XOR converts hypoxanthine to xanthine. GMP forms guanosine, which is subsequently degraded to guanine by purine nucleoside phosphorylase and converted to xanthine by deamination with guanine deaminase. Xanthine is then oxidized by XOR to form the end product UA. Thus, xanthine is converted from hypoxanthine by oxidation with XOR and guanine by deamination with guanine deaminase. The salvage pathway reuses the purine bases and nucleosides obtained from the degradation of AMP and GMP to synthesize new deoxyribonucleic acid and ribonucleic acid. Hypoxanthine and guanine are converted to IMP and GMP by hypoxanthine phosphoribosyltransferase, whereas adenine is converted to AMP by adenine phosphoribosyltransferase and reused to synthesize new deoxyribonucleic acid and ribonucleic acid molecules. Hypoxanthine is salvaged, and xanthine is degraded from guanine in addition to hypoxanthine.

In summary, the UA and xanthine levels were significantly elevated from night to morning, and the rate of increase was positively correlated. These results suggest that the increase in serum UA levels from night to morning may be due to increased UA production. Although both decreased from morning to noon, no correlation was found with the rate of decrease. In healthy men, peak urinary UA excretion has been reported to occur between 08:00 and 10:00 and between 22:00 and 24:00 [36]. Therefore, the decrease from morning to noon may be related to both production and excretion. Furthermore, we suspect that the salvage pathway via the kidneys is the reason for the non-similar results for xanthine and hypoxanthine.

Based on the results for serum UA and plasma xanthine levels and the mechanism of purine metabolism, we expected XOR activity to show similar diurnal variations. However, the XOR activity was significantly elevated from night to morning and was also significantly elevated from morning to noon. XOR is a rate-limiting enzyme in purine metabolism, which mainly catalyzes the conversion of hypoxanthine to xanthine and UA with the concomitant generation of superoxide. XOR activity is increased by hypoxia and various cytokines, such as pro-inflammatory cytokines and tumor necrosis factor [37,38]. In our previous study, XOR activity significantly increased from night to morning in patients with CAD, and the rate of increase in XOR activity was associated with the severity of nocturnal hypoxia. In a hypoxic environment, adenosine triphosphate synthesis is stagnant, thus promoting its decomposition into adenosine diphosphate or AMP, increasing hypoxanthine and xanthine levels, and promoting UA production [39] (Figure 1). Another study on the marine alga Gonyaulax reported that XOR activity is 15 times higher in light than in darkness [40]. Therefore, the increase in XOR activity from night to morning is consistent with previous reports and with the idea that UA production is enhanced at night.

Furthermore, XOR activity increased from morning to noon in the present study. Sun et al. and Kanemitsu et al. reported that XOR activity increases from late light to early dark in rats and in the livers of mice [41,42]. However, both studies indicated that the XOR activity tends to decline after the early dark period. Sun et al. reported a positive correlation between XOR expression and clock genes, indicating a potential functional interplay between the clock gene machinery and purine catabolism [41]. Kanemitsu et al. demonstrated that the anti-hyperuricemic effect of an XOR inhibitor is enhanced by its administration on the day before hepatic XOR activity increased [42]. Few reports are available on circadian variations in XOR activity in animals but not in humans. To the best of our knowledge, this is the first report on its variation in patients with CAD. The morning-to-noon increase in XOR activity in the present study has not been previously reported and differs from the results obtained in rats and mice; therefore, further studies are needed.

This finding suggests that UA production is involved in the increase in UA levels from night to morning. Although XOR activity is speculated to be involved as one of the mechanisms, other triggering factors besides XOR activity may be present. The decrease in UA and xanthine from morning to noon, despite an increase in XOR, suggests that the morning-to-noon variation reflects both production and excretion. Gout attacks occur more frequently during the night and early morning. Based on the results of this study, one possible reason for this may be increased UA production during the night and early morning. Therefore, for clinical application, it is expected that gout attacks can be suppressed by inhibiting XOR activity from night to morning, i.e., by oral administration of XOR inhibitors before sleep. This finding warrants further clinical research.

The present study has several limitations. First, it was a single-arm observational study. This study was conducted only on patients with CAD, and it is necessary to study healthy subjects as a control group. Second, this study was conducted at a single center, and the study population was relatively small in size. However, it is noteworthy that we took measures to determine the appropriate sample size through power analysis, utilizing data from our pilot study involving the initial five subjects. We believe that our findings are reasonable based on previous studies. Third, the inclusion criteria for this study were restricted to male patients only. This decision was influenced by the significant difference in the prevalence of CAD between males and females. Furthermore, female patients with CAD are often older, and a large number of cases is needed to account for age-related bias. Therefore, this study was limited to males. Future studies including females are needed, as the results of this study cannot be applied to females. Fourth, we did not assess UA excretion. Approximately two-thirds of UA is excreted by the kidneys, with the remaining one-third eliminated through the gastrointestinal tract. Future research endeavors should focus on investigating both the production and excretion pathways, utilizing blood, urine, and stool samples. This comprehensive approach will help to further elucidate the mechanisms of diurnal variation in UA, xanthine, and XOR activity. Fifth, we collected samples at only three time points: 18:00, 6:00, and 12:00. These were not equally spaced, and time effects can occur; however, previous reports of diurnal variation in UA have shown a peak at 6:00 and a minimum at 18:00. In addition, to reduce the influence of meals as much as possible, we collected samples at three time points in all patients, at 18:00, 6:00, and 12:00, prior to each meal. Finally, the UA intake was not measured. All patients consumed hospital food; therefore, we considered nutrients and calories within the margin of error. Since individual differences exist in food intake, these differences should be discussed in the results and how they can be interpreted from the perspective of previous studies and of the working hypotheses. The findings and their implications should be discussed in the broadest context possible. Future research directions may also be highlighted.

## 5. Conclusions

Serum UA and plasma xanthine levels showed diurnal variations in patients with CAD. Although XOR activity showed diurnal variation, it was different from that of UA and xanthine. The increase in UA from night to morning was speculated to be due to UA production, whereas the decrease in UA from morning to noon was speculated to be due to both production and excretion.

Furthermore, increased XOR activity from night to morning has been reported to be related to clock genes in addition to hypoxia, which contributes to increased UA production. Gout attacks, which occur more frequently at night and at dawn, may be prevented by suppressing XOR activity before it increases.

## Figures and Tables

**Table 1:** Characteristics of participants.

*n* = 30	
Age, years	67.6 ± 8.2
BMI, kg/m^2^	24.5 ± 3.6
Hypertension, *n* (%)	22 (73)
Dyslipidemia, *n* (%)	27 (90)
Diabetes mellitus, *n* (%)	16 (53)
Current smoker, *n* (%)	8 (27)
Creatinine, mg/dL	0.74 ± 0.12
eGFR, mL/min/1.73 m^2^	83.4 ± 18.4
Medications	
ACEis/ARBs, *n* (%)	16 (53)
β blockers, *n* (%)	21 (70)
Statins, *n* (%)	28 (93)

The values are the means ± s.d. ACEi—angiotensin-converting enzyme inhibitor; ARB—angiotensin II receptor blocker; BMI—body mass index; eGFR—estimated glomerular filtration rate.

**Table 2:** The correlation coefficients between ΔUA and Δxanthine and Δhypoxanthine from 18:00 to 6:00.

	ΔUA		Δxanthine		Δhypoxanthine	
	Coefficient	*p*	Coefficient	*p*	Coefficient	*p*
ΔUA	-	-	0.42	0.02	0.37	0.04
Δxanthine	0.42	0.02	-	-	0.73	<0.001
Δhypoxanthine	0.37	0.04	0.73	<0.001	-	-

UA—uric acid. -—not indicated as the same items are compared.

**Table 3:** The correlation coefficients between ΔUA and Δxanthine and Δhypoxanthine from 6:00 to 12:00.

	ΔUA		Δxanthine		Δhypoxanthine	
	Coefficient	*p*	Coefficient	*p*	Coefficient	*p*
ΔUA	-	-	0.19	0.31	−0.27	0.15
Δxanthine	0.19	0.31	-	-	0.47	<0.01
Δhypoxanthine	−0.27	0.15	0.47	<0.01	-	-

UA—uric acid. -—not indicated as the same items are compared.

## Data Availability

The data supporting the findings of this study are available from the corresponding author upon reasonable request.
